# The global prevalence of dental healthcare needs and unmet dental needs among adolescents: a systematic review and meta-analysis

**DOI:** 10.4178/epih.e2019046

**Published:** 2019-10-27

**Authors:** Mahin Ghafari, Samira Bahadivand-Chegini, Tayebeh Nadi, Amin Doosti-Irani

**Affiliations:** 1 Department of Public Health, School of Health, Shahrekord University of Medical Sciences, Shahrekord, Iran; 2 Department of Epidemiology, School of Public Health, Hamadan University of Medical Sciences, Hamadan, Iran; 3 Research Center for Health Sciences, Hamadan University of Medical Sciences, Hamadan, Iran

**Keywords:** Adolescent, Dental health services, Prevalence, Meta-analysis

## Abstract

**OBJECTIVES:**

Access to dental healthcare services is a major determinant of dental health in communities. This meta-analysis was conducted to estimate the global prevalence of dental needs and of unmet dental needs in adolescents.

**METHODS:**

PubMed, Web of Science, and Scopus were searched in June 2018. The summary measures included the prevalence of met and unmet dental needs. A meta-analysis was performed using the inverse variance method to obtain pooled summary measures. Out of 41,661 retrieved articles, 57 were ultimately included.

**RESULTS:**

The pooled prevalence of orthodontic treatment needs was 46.0% (95% confidence interval [CI], 38.0 to 53.0), that of general treatment needs was 59.0% (95% CI, 42.0 to 75.0), that of periodontal treatment needs was 71.0% (95% CI, 46.0 to 96.0), and that of malocclusion treatment needs was 39.0% (95% CI, 28.0 to 50.0). The pooled prevalence of unmet dental needs was 34.0% (95% CI, 27.0 to 40.0).

**CONCLUSIONS:**

The highest and lowest prevalence of unmet dental needs were found in Southeast Asia and Europe, respectively. The prevalence of dental needs was higher in the countries of the Americas and Europe than in other World Health Organization (WHO) regions. The prevalence of unmet dental needs was higher in Southeast Asia and Africa than in other WHO regions.

## INTRODUCTION

Access to dental healthcare services is a major determinant of dental health in communities. Dental problems, including dental cavities, are most prevalent among adolescents [1].

The global weighted means of decayed, missing, and filled teeth for adolescents aged 12 years old in 2011 and 2015 were 1.67 and 1.86, respectively [2]. In 2010, approximately 2.4 billion people and 621 million children were affected by untreated caries in permanent and deciduous teeth, respectively, and untreated caries in permanent teeth was the most prevalent dental condition worldwide [3]. In the USA, it was reported that 21% of children aged 6-11 years and 58% of adolescents aged 12-19 years had experienced dental caries. In 2011-2012, the prevalence of untreated dental caries was about 6.0% in children and 15.3% in adolescents [4].

Untreated dental cavities have been reported to cause severe pain, infection, and problems with eating, speaking, and learning in children and adolescents [1].

Adolescents constitute a noteworthy age group, as they have specific healthcare needs [5]. Dental healthcare is an important need in this group, given its effects on quality of life and its potential to improve general health. Dental problems remain a huge burden in children and adolescents in certain regions of the world [6]; nevertheless, the prevalence of dental needs in these age groups has not been estimated in some communities [7,8].

Unmet healthcare needs have been defined as the difference between the healthcare services required to cope with a health problem and the services received [9]. Unmet healthcare needs are common in adolescents and are an independent risk factor for health outcomes in adults [10], meaning that they can impose heavy costs on the community, health system, and individuals [7]. Unmet dental needs in adolescents can have consequences that affect quality of life in adulthood. Several studies have been published regarding the prevalence of needs and unmet needs for dental healthcare. However, there are discrepancies in the results of the published studies. The present systematic review and meta-analysis was therefore conducted to estimate the global prevalence of dental needs and unmet dental needs in adolescents by the type of dental care, World Health Organization (WHO) region, and sex.

## MATERIALS AND METHODS

The design of this study is a systematic review and meta-analysis.

As part of a comprehensive systematic review, the present review was conducted to determine the prevalence of dental healthcare needs and unmet dental needs in adolescents. This systematic review was conducted and reported according to the PRISMA (Preferred Reporting Items for Systematic Reviews and Meta-Analyses) [11] (Supplementary Material 1).

### Eligibility criteria

The present review included all retrieved cross-sectional studies that were conducted to estimate dental healthcare needs and unmet dental needs in adolescents. The studies included were not limited regarding the year, location, or language of the study, the sex and race of adolescents studied, or the type of dental health needs and unmet needs studied. According to the WHO, adolescents include individuals aged 10-19 years [12].

Unmet needs were defined as the difference between the healthcare needs present and the healthcare needs that were fulfilled to address the health problems under consideration [9].

### Identifying the relevant studies

The international databases PubMed, Web of Science, and Scopus were searched in June 2018. The keywords used for searching PubMed were as follows: (adolescent [MeSH Terms] OR “teen” [Text Word] OR homeless youth [MeSH Terms] OR “street adolescents” [Text Word]) AND (health services needs and demand [MeSH Terms] OR “unmet needs” [Text Word] OR needs assessment [MeSH Terms] OR “health needs” [Text Word] OR “unmet health needs” [Text Word] OR “health service needs” [Text Word] OR “delay medical care” [Text Word]) AND (oral health [MeSH Terms] OR dental health services [MeSH Terms]). In Web of Science and Scopus, we searched the mentioned keywords as the topic (TS) and TITLE-ABS-KEY, respectively.

### Data extraction and assessing the risk of bias

Endnote X7 software was used to the manage the results of our initial search. Two authors (TN and SBC) were in charge of screening the titles and abstracts of the studies obtained from the databases. The full texts of the selected studies were then evaluated based on the eligibility criteria. Any disagreements between the investigators were resolved through discussion and consultation with a third author (ADI). The kappa value for agreement between two authors in the screening of the title and abstract was 84%.

Three authors (TN, SBC, and ADI) were responsible for data extraction. The data extracted from the included studies comprised the name of the first author, the year of publication, the location (country) of the study, the type of study population, the sex and mean/median age of participants, the type of health need(s) and unmet need(s), the sample size, the number of participants with health needs, and the number of participants with unmet health needs.

Two authors (TN and SBC) were in charge of quality assessments. The Joanna Briggs Institute critical appraisal checklist was used for evaluating the studies that reported prevalence rates and for assessing the risk of bias [13]. The items selected from the Joanna Briggs Institute checklist included (1) the appropriateness of the sampling frame in terms of addressing the target population, (2) the appropriateness of the sampling method, (3) the adequacy of the sample size, (4) the provision of a detailed description of the subjects and the study setting, (5) the use of a valid method for identifying the outcomes (i.e., dental needs and unmet dental needs), (6) the appropriateness of the statistical analysis, and (7) the adequacy of the response rate and the appropriate management of a potential low rate.

### Statistical analysis

The chi-square test was used to examine heterogeneity among the results of the included studies. Between-study variance was assessed using the tau-square test, and the I-square statistic was used to quantify heterogeneity [14].

The summary measures, including the prevalence of dental healthcare needs and unmet dental needs, were extracted from the included studies, and their standard errors were calculated. Meta-analysis was performed using the inverse variance method to obtain the pooled summary measure. In the cases of out-of-range confidence intervals (CIs) in the subgroup analysis, the *metaprop* command was used. A random-effects model was also applied. A p-value of less than 0.05 was considered to indicate statistical significance. The data were analyzed in Review Manager 5.3 (Cochrane Collaboration, Copenhagen, Denmark) and Stata version 11 (StataCorp., College Station, TX, USA).

### Ethics statement

The study protocol was approved by the Ethics Committee of Hamadan University of Medical Sciences (IR.UMSHA.RES.1397.69).

## RESULTS

### Included studies

**Figure 1.**
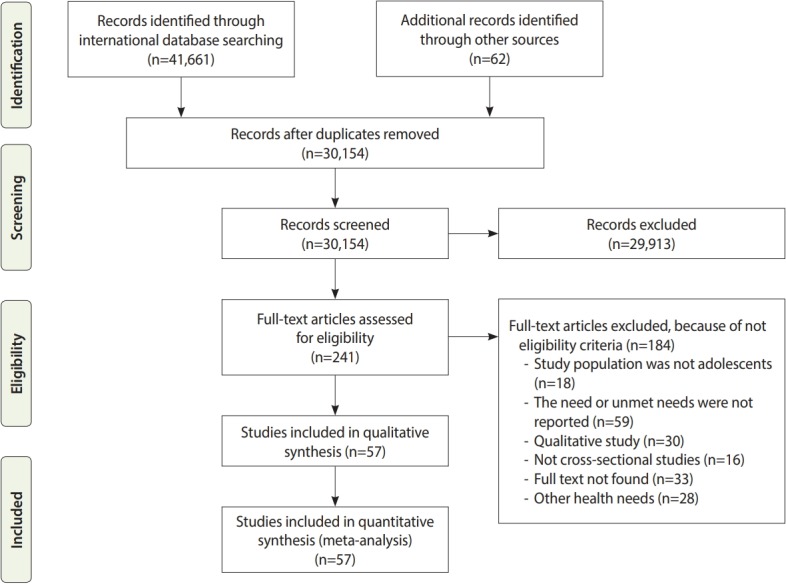
Flowchart depicting the stages through which articles were retrieved and eligibility criteria were checked for the meta-analysis.

**Figure 2.**
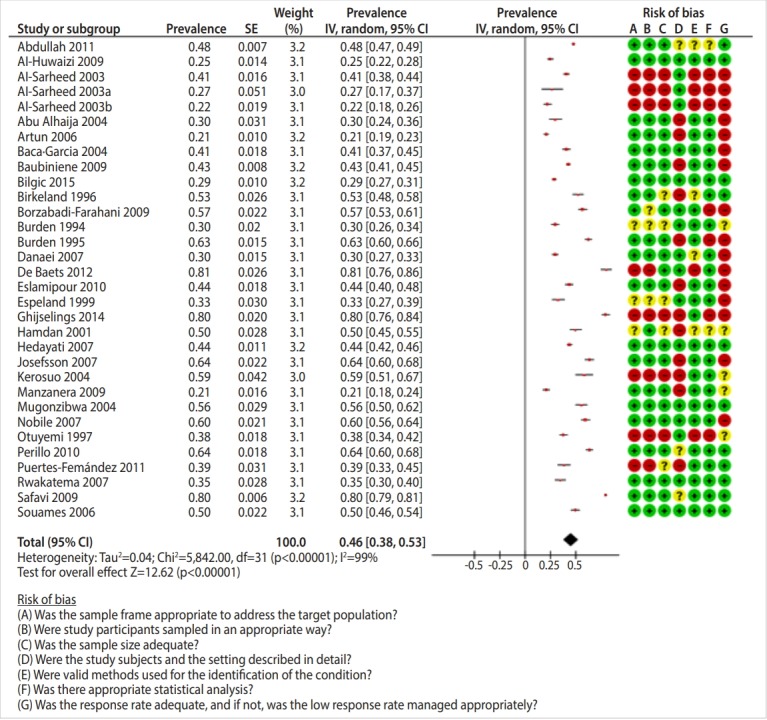
Prevalence of orthodontic treatment needs among adolescents. SE, standard error; CI, confidence interval; df, degree of freedom.

**Figure 3.**
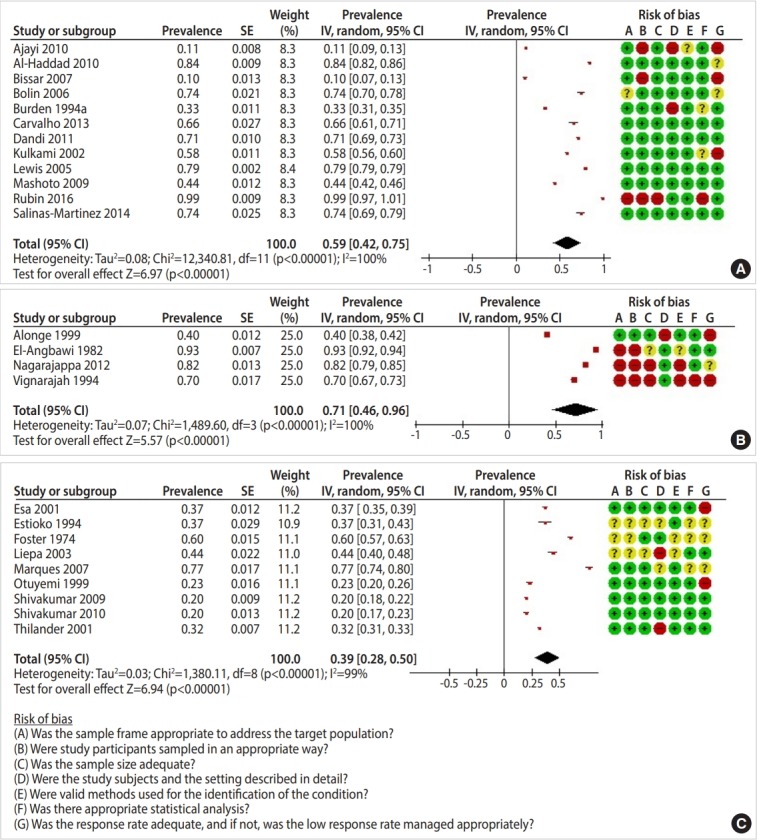
Prevalence of (A) general dental treatment needs, (B) periodontal treatment needs, and (C) malocclusion treatment needs among adolescents. SE, standard error; CI, confidence interval; df, degree of freedom.

**Figure 4.**
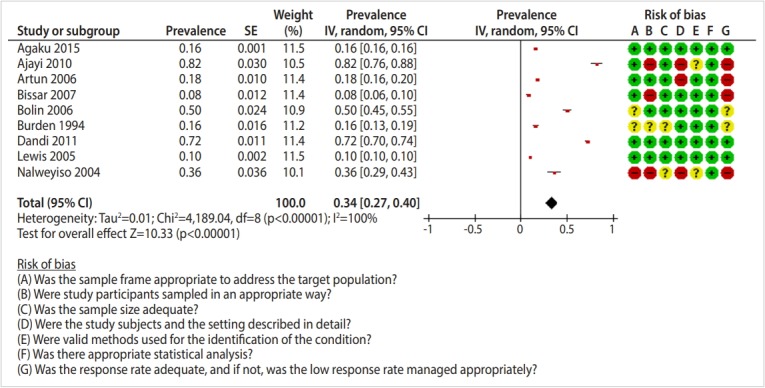
Total prevalence of unmet dental needs among adolescents. SE, standard error; CI, confidence interval; df, degree of freedom.

Out of the 41,661 studies retrieved from searching the international databases and 62 found from scanning the references of the selected studies, 57 studies [15-71] were ultimately included in this systematic review (Figure 1). A study by Al-Sarheed et al. [22] was divided into 3 studies for the purposes of this analysis because it reported dental healthcare needs in 3 groups of adolescents: the general population, visually-impaired adolescents, and adolescents with hearing loss. Table 1 presents the characteristics of the included studies, which included 167,316 adolescents who were evaluated in terms of their dental healthcare needs and 123,821 who were evaluated in terms of their unmet dental healthcare needs. Results of the risk of bias assessment are shown in the forest plots in Figures 2-4.

### Prevalence of dental healthcare needs

The overall prevalence of dental healthcare needs was 49.0% (95% CI, 42.0 to 56.0) across all types of dental care. Table 2 presents the overall prevalence by WHO region, sex, and year of publication of the study. The present review reported the prevalence of each type of dental healthcare need. Orthodontic treatment needs were reported in 54.2% of the studies, general needs in 23.7%, periodontal needs in 6.8%, and malocclusion needs in 12.3%.

The prevalence of orthodontic treatment needs was reported in 32 studies. The pooled prevalence of orthodontic treatment needs was 46.0% (95% CI, 38.0 to 53.0; I^2^=99%) (Figure 2). With regard to WHO region, the highest prevalence was associated with countries in Europe (51.6%; 95% CI, 42.8 to 60.4) and the lowest with countries in Southeast Asia (28.8%; 95% CI, 26.9 to 30.7) (Table 3).

Twelve studies reported the prevalence of general treatment needs in adolescents. The pooled prevalence of general treatment needs was 59.0% (95% CI, 42.0 to 75.0) (Figure 3A). The highest prevalence rates were found in the Eastern Mediterranean region (84.2%; 95% CI, 82.3 to 86.0) and the Africa (78.0%; 95% CI, 77.0 to 80.0). The lowest prevalence was observed in Europe (24.0%; 95% CI, 22.0 to 25.0).

None of the 12 studies were conducted in the Western Pacific region (Table 3).

The pooled prevalence of periodontal treatment needs was 71.0% (95% CI, 46.0 to 96.0) (Figure 3B). The highest prevalence, 93.0% (95% CI, 91.6 to 94.5), was found in the Eastern Mediterranean region (Table 3).

Nine studies reported the prevalence of malocclusion treatment needs in adolescents. The pooled prevalence of this type of need was 39.0% (95% CI, 28.0 to 50.0) (Figure 3C).

### Prevalence of unmet dental healthcare needs

Nine studies reported the prevalence of unmet dental healthcare needs. The pooled prevalence of unmet dental needs was 34.0% (95% CI, 27.0 to 40.0) (Figure 4). The highest prevalence of unmet needs was found in Southeast Asia (72.3%; 95% CI, 70.1 to 74.5) and the lowest in Europe (11.8%; 95% CI, 3.4 to 20.3) (Table 2). Table 3 presents the prevalence of unmet needs by type of dental need and WHO region.

## DISCUSSION

According to the results of the present systematic review, dental healthcare is a major global need in adolescents. Across all types of dental care, about 50% of adolescents worldwide were found to require dental healthcare services, and 34.0% were found to have unmet dental healthcare needs. The highest prevalence of these needs was observed in countries in the Americas and Europe, and the lowest was seen in Africa and the Western Pacific region. The seemingly higher prevalence observed in the Americas and Europe compared to Africa and the Western Pacific can be explained by the lower number of studies conducted in developing countries and their lower sample sizes compared to studies conducted in developed countries. The larger number of studies conducted on dental healthcare in developed countries suggests the greater perceived importance of dental health among adolescents in these countries. Developed countries therefore appear to have made more serious efforts than developing countries to identify dental health problems in adolescents.

In contrast, the prevalence of unmet dental healthcare needs was lower in Europe and the Americas than in the other regions of the WHO. This prevalence was higher in Southeast Asia and Africa than in the other regions. Unmet dental needs therefore appear to be mainly associated with developing countries. In low-income and middle-income countries, the cost of dental healthcare can put a substantial financial burden on households [72]. In addition, members of the general public in these countries are not adequately protected against the high costs of dental healthcare [72]. A study conducted in Iran showed that the cost of essential dental care was an important determinant of catastrophic healthcare expenditures [73]. The high expenditures required for dental healthcare and the lack of associated insurance coverage in many countries, especially low-income and middle-income countries, can contribute to the high prevalence of unmet dental healthcare needs in these countries.

Globally, unmet dental needs are common in adolescents. Unmet dental needs are an independent risk factor for oral health outcomes in adulthood [10], meaning that they can impose a high burden on the community, health system, and individuals [7]. Therefore, addressing unmet dental needs is important in terms of public health. Unmet dental needs affect the dental health-associated quality of life in adolescents [74]. Improving dental healthcare services and meeting dental healthcare needs can therefore promote overall quality of life in adolescents; nevertheless, given the high expenditures required for dental healthcare, policy-makers are recommended to more effectively provide households with the financial support they need for this highly expensive care. Moreover, the total number of people with unmet oral healthcare needs increased from 2.5 billion in 1990 to 3.5 billion in 2015, suggesting that oral health remains a global public health challenge [75], as emphasized by the global results of the present study in adolescents.

Worldwide, there is a lack of knowledge about certain types of dental healthcare needs; for instance, no compelling evidence was found regarding the global prevalence of unmet periodontal and malocclusion treatment needs. The lack of knowledge regarding the prevalence of unmet dental healthcare needs is more serious at a global than at a local scale. The 9 studies included in the present review regarding unmet dental needs were limited to unmet general and orthodontic dental treatment needs. We therefore recommend that further studies be conducted on unmet dental healthcare needs in adolescents, especially in low-income and middle-income countries.

The present systematic review and meta-analysis was faced with high heterogeneity between the results obtained in the included studies. Homogeneity was not achieved, despite conducting the subgroup analysis by WHO region and type of dental healthcare. The high heterogeneity observed can be explained by differences in the setting, time, and location of studies, in the type of dental healthcare, in the methods of evaluating of dental health needs and unmet needs, and in the quality of the included studies.

The major limitations of the present systematic review and meta-analysis included the low quality of some of the included studies and their use of different tools and criteria for detecting dental healthcare needs. In addition, our results may be affected by selection bias due to lack of access to the full text of some papers as well as the potential existence of studies in the gray literature, such as theses and annual unpublished reports by nations regarding the prevalence of need and unmet needs.

## CONCLUSION

The results obtained from this systematic review suggest that the prevalence of dental healthcare needs and unmet dental healthcare needs is globally significant in adolescents. The prevalence of dental healthcare needs was higher in the countries of the Americas and Europe than in other WHO regions. Unmet needs were more prevalent in Southeast Asia and Africa than in other WHO regions.

## Figures and Tables

**Table 1.** Characteristics of included studies

Study	Country	WHO region	Study population	Age (yr)	Sex	Sample size (n)	Need	Unmet	Type of dental health need(s)
Bilgic et al., 2015 [26]	Turkey	Southeast Asia	General	12-16	Both	2,250	648		Orthodontic treatment
									DHC
Bolin et al., 2006 [29]	USA	Americas	Adolescents in a juvenile detention facility	12-17	Both	419	310	208	Overall dental treatment needs
									Dental caries
Vignarajah, 1994 [71]	Antigua and Barbuda	Americas	General	12-19	Both	702	494		Periodontal treatment needs
De Baets et al., 2012 [37]	Belgium	European	General	11-16	Female	223	180		Orthodontic treatment
									DHC
Agaku et al., 2015 [16]	USA	Americas	General	6-17	Both	65,593		10,338	Overall dental treatment needs
									Appropriate and timely preventive or therapeutic dental healthcare
Kulkami et al., 2002 [49]	India	Southeast Asia	General	11-15	Both	2005	1,159		Overall dental treatment needs
									Dental caries
Ajayi et al., 2010 [17]	Nigeria	African	General	12-19	Both	1,532	165	135	Traumatized treatment
									Traumatized anterior teeth
Ghijselings et al., 2014 [44]	Belgium	European	General	11-16	Both	386	310		Orthodontic treatment
									DHC
Al-Haddad et al., 2010 [18]	Yemen	Eastern Mediterranean	General	6-14	Both	1,489	1,253		Overall dental treatment needs
Nagarajappa et al., 2012 [56]	India	Southeast Asia	General	12-15	Both	900	740		Periodontal treatment needs
Al-Huwaizi et al., 2009 [20]	Iraq	Eastern Mediterranean	General	13	Both	998	413		Orthodontic treatment
									DAI
Bissar et al., 2007 [28]	Germany	European	General	11-13	Both	502	51	38	Overall dental treatment needs
									Restorative treatment need
Otuyemi et al., 1997 [60]	Nigeria	African	General	12-18	Both	704	271		Orthodontic treatment
									DHC
Rubin et al., 2016 [63]	Uganda	African	General	5-17	Female	153	151		Overall dental treatment needs
Borzabadi-Farahani et al., 2009 [30]	Iran	Eastern Mediterranean	General	11-14	Both	496	281		Orthodontic treatment
									DHC
Alonge et al., 1999 [21]	Saint Vincent and the Grenadines	Americas	General	7-15	Both	1,646	662		Periodontal treatment needs
Safavi et al., 2009 [65]	Iran	Eastern Mediterranean	General	14-16	Both	5,091	4,079		Orthodontic treatment
									DHC
Burden et al., 1994 [31]	Northern Ireland	European	General	15-16	Both	506	154	82	Orthodontic treatment
Salinas-Martínez et al., 2014 [66]	Mexico	Americas	General	13	Both	301	223		Overall dental treatment needs
Al-Sarheed et al., 2003 [22]	Saudi Arabia	Eastern Mediterranean	Visually impaired adolescents	11-16	Both	77	21		Orthodontic treatment
									DHC
Al-Sarheed et al., 2003 [22]	Saudi Arabia	Eastern Mediterranean	Hearing-impaired adolescents	11-16	Both	210	62		Orthodontic treatment
									DHC
Al-Sarheed et al., 2003 [22]	Saudi Arabia	Eastern Mediterranean	General	11-16	Both	494	108		Orthodontic treatment
									DHC
Carvalho et al., 2013 [34]	Brazil	Americas	General	12-14	Both	300	198		Overall dental treatment needs
									Dental caries
Danaei et al., 2007 [35]	Iran	Eastern Mediterranean	General	12-15	Both	900	269		Orthodontic treatment
									DAI
Dandi et al., 2011 [36]	India	Southeast Asia	General	12	Both	2,203	1,573	1,137	Overall dental treatment needs
									Dental pain
Artun et al., 2006 [23]	Kuwait	Eastern Mediterranean	General	13-14	Both	1,583	330	290	Orthodontic treatment
El-Angbawi et al., 1982 [38]	Saudi Arabia	Eastern Mediterranean	General	13-15	Both	1,174	1,092		Periodontal treatment needs
Baubiniene et al., 2009 [25]	Lithuania	European	General	10-15	Male	4,235	1,806		Orthodontic treatment
Eslamipour et al., 2010 [40]	Iran	Eastern Mediterranean	General	11-20	Both	748	331		Orthodontic treatment
									DAI
Abdullah et al., 2001 [15]	Malaysia	Western Pacific	General	12-13	Both	5,112	2,449		Orthodontic treatment
									DHC
Abu Alhaija et al., 2004 [19]	Jordan	Eastern Mediterranean	General	12-14	Both	1,002	252		Orthodontic treatment
									DHC
Baca-Garcia et al., 2004 [24]	Spain	European	General	14-20	Both	744	308		Orthodontic treatment
									DAI
Birkeland et al., 1996 [27]	Norway	European	General	11	Both	359	191		Orthodontic treatment
									DAI
Burden et al., 1994 [33]	UK	European	General	11-12	Both	1,829	600		Overall dental treatment needs
Burden et al., 1995 [32]	Ireland	European	General	11-12	Both	1,107	697		Orthodontic treatment
Esa et al., 2001 [39]	Malaysia	Western Pacific	General	12-13	Both	1,519	566		Malocclusion and orthodontic treatment need
Espeland et al., 1999 [41]	Norway	European	General	16-20	Both	250	82		Orthodontic treatment
Estioko et al., 1994 [42]	Australia	Western Pacific	General	12-16	Both	268	98		Malocclusion and orthodontic treatment need
Foster et al., 1974 [43]	UK	European	General	11-12	Both	1,000	599		Malocclusion and orthodontic treatment need
Hamdan., 2001 [45]	Jordan	Eastern Mediterranean	General	14-17	Both	320	160		Orthodontic treatment
									DHC
Hedayati et al., 2007 [46]	Iran	Eastern Mediterranean	General	11-14	Both	1,965	869		Orthodontic treatment
									DHC
Josefsson et al., 2007 [47]	Sweden	European	General	12-13	Both	476	307		Orthodontic treatment
									DHC
Kerosuo et al., 2004 [48]	Kuwait	Eastern Mediterranean	General	14-18	Both	139	82		Orthodontic treatment
									DHC
Lewis et al., 2005 [50]	USA	Americas	Children with special healthcare needs	≤17	Both	38,866	30,815	3,205	Overall dental treatment needs
Liepa et al., 2003 [51]	Latvia	European	General	12-13	Both	505	222		Malocclusion and orthodontic treatment need
Manzanera et al., 2009 [52]	Spain	European	General	12-16	Both	655	139		Orthodontic treatment
									DHC
Marques et al., 2007 [53]	Brazil	Americas	General	13-15	Both	600	462		Malocclusion and orthodontic treatment need DAI
Mashoto et al., 2009 [54]	Tanzania	African	General	10-19	Both	1,780	790		Overall dental treatment needs
Mugonzibwa et al., 2004 [55]	Tanzania	African	General	9-18	Both	295	164		Orthodontic treatment
									DHC
Nalweyiso et al., 2004 [57]	Uganda	African	General	12	Both	181		65	Overall dental treatment needs
Nobile et al., 2007 [58]	Italy	European	General	11-15	Both	546	325		Orthodontic treatment
									DHC
Otuyemi et al., 1997 [60]	Nigeria	African	General	12-18	Both	703	159		Malocclusion and orthodontic treatment need
Perillo et al., 2010 [61]	Italy	European	General	12	Both	703	451		Orthodontic treatment
									DHC
Puertes-Fernández et al., 2011 [62]	Spain	European	General	12	Both	248	97		Orthodontic treatment
									DHC
Rwakatema et al., 2007 [64]	Tanzania	African	General	12-15	Both	289	102		Orthodontic treatment
									DAI
Shivakumar et al., 2009 [67]	India	Southeast Asia	General	12-15	Both	1,000	199		Malocclusion and orthodontic treatment need
Shivakumar et al., 2010 [68]	India	Southeast Asia	General	12-15	Both	1,800	362		Malocclusion and orthodontic treatment need
Souames et al., 2006 [69]	France	European	General	9-12	Both	511	255		Orthodontic treatment
									DHC
Thilander et al., 2001 [70]	Colombia	Americas	General	5-17	Both	4,724	1,504		Malocclusion and orthodontic treatment need

WHO, World Health Organization; DHC, dental health component; DAI, Dental Aesthetic Index.

**Table 2.** Prevalence of any dental healthcare need and unmet needs among adolescents based on WHO region and sex

Variables		Need				Unmet need			
		n	Prevalence (95% CI)	I^2^	p-value	n	Prevalence (95% CI)	I^2^	p-value
WHO region	Americas	8	64.1 (45.3, 82.8)	99.9	<0.001	3	23.2 (18.0, 28.5)	99.7	<0.001
	Southeast Asia	6	46.7 (25.4, 68.0)	99.8	<0.001	1	72.3 (70.1, 74.5)	-	-
	African	7	34.4 (19.5, 49.4)	99.4	<0.001	2	58.9 (13.9, 100)	99.0	<0.001
	European	18	43.7 (13.7, 73.7)	99.6	<0.001	2	11.8 (3.4, 20.3)	94.5	<0.001
	Eastern Mediterranean	15	47.2 (32.6, 61.8)	99.8	<0.001	1	18.3 (16.4, 20.2)	-	-
	Western Pacific	3	40.8 (32.3, 49.3)	96.9	<0.001	-	-	-	-
Sex	Male	19	50.0 (37.5, 63.5)	99.7	<0.001	3	37.9 (4.6, 71.2)	99.9	<0.001
	Female	21	49.8 (36.8, 62.9)	99.7	<0.001	3	33.8 (29.0, 38.7)	99.8	<0.001
	Both	33	47.9 (38.4, 57.3)	99.8	<0.001	6	33.3 (19.1, 47.6)	99.4	<0.001
Year (range)	1974-1999	12	47.8 (31.8, 63.8)	99.7	<0.001	1	16.2 (13.0, 19.4)	-	-
	2000-2004	13	40.1 (34.2, 47.1)	98.6	<0.001	1	35.9 (28.9, 42.9)	-	-
	2005-2009	19	48.2 (36.5, 59.9)	99.9	<0.001	4	21.1 (11.9, 30.3)	99.1	<0.001
	2010-2016	13	60.0 (41.0, 79.1)	99.9	<0.001	3	56.6 (10.3, 99.0)	99.8	<0.001
Sample size (n)	≤500	19	54.5 (41.9, 67.2)	99.3	<0.001	2	43.0 (29.6, 56.5)	90.1	<0.001
	501-1,000	18	44.8 (34.1, 55.5)	99.5	<0.001	2	11.8 (3.40, 20.3)	94.5	<0.001
	≥1,001	20	47.8 (36.0, 59.6)	99.9	<0.001	5	38.9 (30.5, 47.2)	99.9	<0.001
Total		57	49.0 (42.0, 56.0)	99.9	<0.001	9	34.0 (27.0, 40.0)	99.9	<0.001

WHO, World Health Organization; CI, confidence interval.

**Table 3.** Prevalence of specific dental healthcare needs and unmet needs among adolescents based on WHO region

Variables		Need			Unmet need		
		n	Prevalence (95% CI)	I^2^	n	Prevalence (95% CI)	I^2^
Orthodontic treatment	Americas	-	-	-	-	-	-
	Southeast Asia	1	28.8 (26.9, 30.7)	-	-	-	-
	African	3	43.0 (31.9, 54.2)	93.6	-	-	-
	European	14	51.6 (42.8, 60.4)	98.9	1	16.2 (13.0, 19.4)	-
	Eastern Mediterranean	13	40.8 (25.6, 56.0)	99.7	1	18.3 (16.4, 20.2)	-
	Western Pacific	1	47.9 (46.5, 49.3)	-	-	-	-
General dental treatment needs	Americas	4	73.7 (67.9, 79.5)	90.1	3	23.2 (18.0, 28.5)	99.7
	Southeast Asia	2	64.6 (51.3, 77.9)	98.8	1	72.3 (70.1, 74.5)	-
	African	3	78.0 (77.0, 80.0)	99.8	2	58.9 (13.9, 100)	99.0
	European	2	24.0 (22.0, 25.0)	99.9	1	7.6 (5.3, 9.9)	-
	Eastern Mediterranean	1	84.2 (82.3, 86.0)	-	-	-	-
	Western Pacific	-	-	-	-	-	-
Malocclusion treatment	Americas	2	54.4 (10.1, 98.6)	99.8	-	-	-
	Southeast Asia	2	20.0 (18.6, 21.5)	00.0	-	-	-
	African	1	22.6 (19.5, 25.7)	-	-	-	-
	European	2	52.0 (36.4, 67.6)	97.1	-	-	-
	Eastern Mediterranean	-	-	-	-	-	-
	Western Pacific	2	37.2 (34.9, 39.4)	00.0	-	-	-
Periodontal treatment needs	Americas	2	55.3 (25.7, 84.8)	99.5	-	-	-
	Southeast Asia	1	82.2 (79.7, 84.7)	-	-	-	-
	African	-	-	-	-	-	-
	European	-	-	-	-	-	-
	Eastern Mediterranean	1	93.0 (91.6, 94.5)	-	-	-	-
	Western Pacific	-	-	-			

WHO, World Health Organization region; CI, confidence interval.

## References

[1] Centers for Disease Control and Prevention (2017). Children’s dental health. https://www.cdc.gov/features/childrens-dental-health/index.html.

[2] World Health Organization Oral health databases. https://www.who.int/oral_health/databases/en/.

[3] Kassebaum NJ, Bernabé E, Dahiya M, Bhandari B, Murray CJ, Marcenes W (2015). Global burden of untreated caries: a systematic review and metaregression. J Dent Res.

[4] Dye BA, Thornton-Evans G, Li X, Iafolla TJ (2015). Dental caries and sealant prevalence in children and adolescents in the United States, 2011-2012. NCHS Data Brief.

[5] World Health Organization Adolescent responsive health systems. https://www.who.int/maternal_child_adolescent/topics/adolescence/health_services/en/.

[6] Wang Z, Deng Y, Liu SW, He J, Ji K, Zeng XY (2017). Prevalence and years of life lost due to disability from dental caries among children and adolescents in Western China, 1990–2015. Biomed Environ Sci.

[7] Marshall EG (2011). Do young adults have unmet healthcare needs?. J Adolesc Health.

[8] Sawyer SM, McNeil R, McCarthy M, Orme L, Thompson K, Drew S (2017). Unmet need for healthcare services in adolescents and young adults with cancer and their parent carers. Support Care Cancer.

[9] Sanmartin C, Houle C, Tremblay S, Berthelot JM (2002). Changes in unmet health care needs. Health Rep.

[10] Hargreaves DS, Elliott MN, Viner RM, Richmond TK, Schuster MA (2015). Unmet health care need in US adolescents and adult health outcomes. Pediatrics.

[11] Moher D, Liberati A, Tetzlaff J, Altman DG; PRISMA Group (2009). Preferred reporting items for systematic reviews and meta-analyses: the PRISMA statement. PLoS Med.

[12] World Health Organization Recognizing adolescence. http://apps.who.int/adolescent/second-decade/section2/page1/recognizing-adolescence.html.

[13] Joanna Briggs Institute (JBI) (2017). Critical appraisal tools for use in JBI systematic reviews checklist for prevalence studies. https://joannabriggs.org/sites/default/files/2019-05/JBI_Critical_Appraisal-Checklist_for_Prevalence_Studies2017_0.pdf.

[14] Higgins JP, Thompson SG, Deeks JJ, Altman DG (2003). Measuring inconsistency in meta-analyses. BMJ.

[15] Abdullah MS, Rock WP (2001). Assessment of orthodontic treatment need in 5,112 Malaysian children using the IOTN and DAI indices. Community Dent Health.

[16] Agaku IT, Olutola BG, Adisa AO, Obadan EM, Vardavas CI (2015). Association between unmet dental needs and school absenteeism because of illness or injury among U.S. school children and adolescents aged 6-17 years, 2011-2012. Prev Med.

[17] Ajayi MD, Denloye O, Abiodun Solanke FI (2010). The unmet treatment need of traumatized anterior teeth in selected secondary school children in Ibadan, Nigeria. Dent Traumatol.

[18] Al-Haddad KA, Al-Hebshi NN, Al-Ak’hali MS (2010). Oral health status and treatment needs among school children in Sana’a City, Yemen. Int J Dent Hyg.

[19] Abu Alhaija ES, Al-Nimri KS, Al-Khateeb SN (2004). Orthodontic treatment need and demand in 12-14-year-old north Jordanian school children. Eur J Orthod.

[20] Al-Huwaizi AF, Rasheed TA (2009). Assessment of orthodontic treatment needs of Iraqi Kurdish teenagers using the Dental Aesthetic Index. East Mediterr Health J.

[21] Alonge OK, Narendran S (1999). Periodontal health status of school children in St. Vincent and the Grenadines. Odontostomatol Trop.

[22] Al-Sarheed M, Bedi R, Hunt NP (2003). Orthodontic treatment need and self-perception of 11-16-year-old Saudi Arabian children with a sensory impairment attending special schools. J Orthod.

[23] Artun J, Kerosuo H, Behbehani F, Al-Jame B (2006). Residual need for early orthodontic treatment and orthodontic treatment experience among 13- to 14-year-old school children in Kuwait. Med Princ Pract.

[24] Baca-Garcia A, Bravo M, Baca P, Baca A, Junco P (2004). Malocclusions and orthodontic treatment needs in a group of Spanish adolescents using the Dental Aesthetic Index. Int Dent J.

[25] Baubiniene D, Sidlauskas A, Miseviciene I (2009). The need for orthodontic treatment among 10-11- and 14-15-year-old Lithuanian schoolchildren. Medicina (Kaunas).

[26] Bilgic F, Gelgor IE, Celebi AA (2015). Malocclusion prevalence and orthodontic treatment need in central Anatolian adolescents compared to European and other nations’ adolescents. Dental Press J Orthod.

[27] Birkeland K, Boe OE, Wisth PJ (1996). Orthodontic concern among 11- year-old children and their parents compared with orthodontic treatment need assessed by index of orthodontic treatment need. Am J Orthod Dentofacial Orthop.

[28] Bissar AR, Oikonomou C, Koch MJ, Schulte AG (2007). Dental health, received care, and treatment needs in 11- to 13-year-old children with immigrant background in Heidelberg, Germany. Int J Paediatr Dent.

[29] Bolin K, Jones D (2006). Oral health needs of adolescents in a juvenile detention facility. J Adolesc Health.

[30] Borzabadi-Farahani A, Borzabadi-Farahani A, Eslamipour F (2009). Orthodontic treatment needs in an urban Iranian population, an epidemiological study of 11-14 year old children. Eur J Paediatr Dent.

[31] Burden DJ, Holmes A (1994). The need for orthodontic treatment in the child population of the United Kingdom. Eur J Orthod.

[32] Burden DJ (1995). Need for orthodontic treatment in Northern Ireland. Community Dent Oral Epidemiol.

[33] Burden DJ, Mitropoulos CM, Shaw WC (1994). Residual orthodontic treatment need in a sample of 15- and 16-year-olds. Br Dent J.

[34] Carvalho JC, Rebelo MA, Vettore MV (2013). The relationship between oral health education and quality of life in adolescents. Int J Paediatr Dent.

[35] Danaei SM, Amirrad F, Salehi P (2007). Orthodontic treatment needs of 12-15-year-old students in Shiraz, Islamic Republic of Iran. East Mediterr Health J.

[36] Dandi KK, Rao EV, Margabandhu S (2011). Dental pain as a determinant of expressed need for dental care among 12-year-old school children in India. Indian J Dent Res.

[37] De Baets E, Lambrechts H, Lemiere J, Diya L, Willems G (2012). Impact of self-esteem on the relationship between orthodontic treatment need and oral health-related quality of life in 11- to 16-year-old children. Eur J Orthod.

[38] El-Angbawi MF, Younes SA (1982). Periodontal disease prevalence and dental needs among schoolchildren in Saudi Arabia. Community Dent Oral Epidemiol.

[39] Esa R, Razak IA, Allister JH (2001). Epidemiology of malocclusion and orthodontic treatment need of 12-13-year-old Malaysian schoolchildren. Community Dent Health.

[40] Eslamipour F, Borzabadi-Farahani A, Asgari I (2010). Assessment of orthodontic treatment need in 11- to 20-year-old urban Iranian children using the Dental Aesthetic Index (DAI). World J Orthod.

[41] Espeland L, Stenvik A (1999). Residual need in orthodontically untreated 16-20-year-olds from areas with different treatment rates. Eur J Orthod.

[42] Estioko LJ, Wright FA, Morgan MV (1994). Orthodontic treatment need of secondary schoolchildren in Heidelberg, Victoria: an epidemiologic study using the Dental Aesthetic Index. Community Dent Health.

[43] Foster TD, Day AJ (1974). A survey of malocclusion and the need for orthodontic treatment in a Shropshire school population. Br J Orthod.

[44] Ghijselings I, Brosens V, Willems G, Fieuws S, Clijmans M, Lemiere J (2014). Normative and self-perceived orthodontic treatment need in 11- to 16-year-old children. Eur J Orthod.

[45] Hamdan AM (2001). Orthodontic treatment need in Jordanian school children. Community Dent Health.

[46] Hedayati Z, Fattahi HR, Jahromi SB (2007). The use of index of orthodontic treatment need in an Iranian population. J Indian Soc Pedod Prev Dent.

[47] Josefsson E, Bjerklin K, Lindsten R (2007). Malocclusion frequency in Swedish and immigrant adolescents--influence of origin on orthodontic treatment need. Eur J Orthod.

[48] Kerosuo H, Al Enezi S, Kerosuo E, Abdulkarim E (2004). Association between normative and self-perceived orthodontic treatment need among Arab high school students. Am J Orthod Dentofacial Orthop.

[49] Kulkami SS, Deshpande SD (2002). Caries prevalence and treatment needs in 11-15 year old children of Belgaum city. J Indian Soc Pedod Prev Dent.

[50] Lewis C, Robertson AS, Phelps S (2005). Unmet dental care needs among children with special health care needs: implications for the medical home. Pediatrics.

[51] Liepa A, Urtane I, Richmond S, Dunstan F (2003). Orthodontic treatment need in Latvia. Eur J Orthod.

[52] Manzanera D, Montiel-Company JM, Almerich-Silla JM, Gandía JL (2009). Orthodontic treatment need in Spanish schoolchildren: an epidemiological study using the Index of Orthodontic Treatment Need. Eur J Orthod.

[53] Marques CR, Couto GB, Orestes Cardoso S (2007). Assessment of orthodontic treatment needs in Brazilian schoolchildren according to the Dental Aesthetic Index (DAI). Community Dent Health.

[54] Mashoto KO, Åstrøm AN, David J, Masalu JR (2009). Dental pain, oral impacts and perceived need for dental treatment in Tanzanian school students: a cross-sectional study. Health Qual Life Outcomes.

[55] Mugonzibwa EA, Kuijpers-Jagtman AM, Van ‘t Hof MA, Kikwilu EN (2004). Perceptions of dental attractiveness and orthodontic treatment need among Tanzanian children. Am J Orthod Dentofacial Orthop.

[56] Nagarajappa R, Kenchappa M, Ramesh G, Nagarajappa S, Tak M (2012). Assessment of periodontal status and treatment needs among 12 and 15 years old school children in Udaipur, India. Eur Arch Paediatr Dent.

[57] Nalweyiso N, Busingye J, Whitworth J, Robinson PG (2004). Dental treatment needs of children in a rural subcounty of Uganda. Int J Paediatr Dent.

[58] Nobile CG, Pavia M, Fortunato L, Angelillo IF (2007). Prevalence and factors related to malocclusion and orthodontic treatment need in children and adolescents in Italy. Eur J Public Health.

[59] Otuyemi OD, Ogunyinka A, Dosumu O, Cons NC, Jenny J (1999). Malocclusion and orthodontic treatment need of secondary school students in Nigeria according to the dental aesthetic index (DAI). Int Dent J.

[60] Otuyemi OD, Ugboko VI, Adekoya-Sofowora CA, Ndukwe KC (1997). Unmet orthodontic treatment need in rural Nigerian adolescents. Community Dent Oral Epidemiol.

[61] Perillo L, Masucci C, Ferro F, Apicella D, Baccetti T (2010). Prevalence of orthodontic treatment need in southern Italian schoolchildren. Eur J Orthod.

[62] Puertes-Fernández N, Montiel-Company JM, Almerich-Silla JM, Manzanera D (2011). Orthodontic treatment need in a 12-year-old population in the Western Sahara. Eur J Orthod.

[63] Rubin PF, Winocur E, Erez A, Birenboim-Wilensky R, Peretz B (2016). Dental treatment needs among children and adolescents residing in an Ugandan Orphanage. J Clin Pediatr Dent.

[64] Rwakatema DS, Kemoli AM (2007). Orthodontic treatment needs amont 12-15 year-olds in Moshi, Tanzania. East Afr Med J.

[65] Safavi SM, Sefidroodi A, Nouri M, Eslamian L, Kheirieh S, Bagheban AA (2009). Orthodontic treatment need in 14-16 year-old Tehran high school students. Aust Orthod J.

[66] Salinas-Martínez AM, Hernández-Elizondo RT, Núñez-Rocha GM, Ramos Peña EG (2014). Psychometric properties of the Spanish version of the short-form Child Perceptions Questionnaire for 11-14-year-olds for assessing oral health needs of children. J Public Health Dent.

[67] Shivakumar KM, Chandu GN, Subba Reddy VV, Shafiulla MD (2009). Prevalence of malocclusion and orthodontic treatment needs among middle and high school children of Davangere city, India by using Dental Aesthetic Index. J Indian Soc Pedod Prev Dent.

[68] Shivakumar K, Chandu G, Shafiulla M (2010). Severity of malocclusion and orthodontic treatment needs among 12- to 15-year-old school children of Davangere District, Karnataka, India. Eur J Dent.

[69] Souames M, Bassigny F, Zenati N, Riordan PJ, Boy-Lefevre ML (2006). Orthodontic treatment need in Spanish schoolchildren: an epidemiological study using the Index of Orthodontic Treatment Need. Eur J Orthod.

[70] Thilander B, Pena L, Infante C, Parada SS, de Mayorga C (2001). Prevalence of malocclusion and orthodontic treatment need in children and adolescents in Bogota, Colombia. An epidemiological study related to different stages of dental development. Eur J Orthod.

[71] Vignarajah S (1994). Periodontal treatment needs in 12 and 15 to 19-year-old school children in the Caribbean Island of Antigua, 1990. J Periodontal Res.

[72] Masood M, Sheiham A, Bernabé E (2015). Household expenditure for dental care in low and middle income countries. PLoS One.

[73] Kavosi Z, Rashidian A, Pourreza A, Majdzadeh R, Pourmalek F, Hosseinpour AR (2012). Inequality in household catastrophic health care expenditure in a low-income society of Iran. Health Policy Plan.

[74] Kunz F, Platte P, Keß S, Geim L, Zeman F, Proff P (2018). Correlation between oral health-related quality of life and orthodontic treatment need in children and adolescents-a prospective interdisciplinary multicentre cohort study. J Orofac Orthop.

[75] Kassebaum NJ, Smith AG, Bernabé E, Fleming TD, Reynolds AE, Vos T (2017). Global, regional, and national prevalence, incidence, and disability-adjusted life years for oral conditions for 195 countries, 1990-2015: a systematic analysis for the global burden of diseases, injuries, and risk factors. J Dent Res.

